# Integrative descriptions of two new species of *Dugesia* from Hainan Island, China (Platyhelminthes, Tricladida, Dugesiidae)

**DOI:** 10.3897/zookeys.1028.60838

**Published:** 2021-04-05

**Authors:** Lei Wang, Zi-mei Dong, Guang-wen Chen, Ronald Sluys, De-zeng Liu

**Affiliations:** 1 College of Life Science, Henan Normal University, Xinxiang, 453007 Henan, China Henan Normal University Xinxiang China; 2 Naturalis Biodiversity Center, Leiden, the Netherlands Xinxiang University Xinxiang China; 3 Medical College, Xinxiang University, Xinxiang 453003, China Naturalis Biodiversity Center Leiden Netherlands

**Keywords:** *Histology*, karyology, molecular distance, molecular phylogeny, taxonomy

## Abstract

Two new species of the genus *Dugesia* (Platyhelminthes, Tricladida, Dugesiidae) from Hainan Island of China are described on the basis of morphological, karyological and molecular data. *Dugesia semiglobosa* Chen & Dong, **sp. nov**. is mainly characterized by a hemispherical, asymmetrical penis papilla with ventrally displaced ejaculatory duct opening terminally at tip of penis papilla; vasa deferentia separately opening into mid-dorsal portion of intrabulbar seminal vesicle; two diaphragms in the ejaculatory duct; copulatory bursa formed by expansion of bursal canal, lined with complex stratified epithelium, which projects through opening in bursa towards intestine, without having open communication with the gut; mixoploid chromosome complement diploid (2n = 16) and triploid (3n = 24), with metacentric chromosomes. *Dugesia majuscula* Chen & Dong, **sp. nov**. is mainly characterized by oviducts opening asymmetrically into female reproductive system; hyperplasic ovaries; expanded posterior section of bursal canal; vasa deferentia separately opening into mid-dorsal portion of seminal vesicle; asymmetrical penis papilla due to ventral course of ejaculatory duct, which has subterminal and dorsal opening at tip papilla; mixoploid chromosome complement diploid (2n = 16) and triploid (3n = 24); chromosomes metacentric. Apart from their anatomy, separate species status of the two new species is supported also by their genetic distances and by their positions in the phylogenetic tree. The sexualization process may have been induced by the lower temperatures, in comparison with their natural habitat, under which the worms were cultured in the laboratory.

## Introduction

Approximately 94 species of the freshwater planarian genus *Dugesia* Girard, 1850 have been reported from a major portion of the Old World and Australia (Sluys and Riutort 2018; Song et al. 2020). Thus far, 22 species of *Dugesia* are known from the Oriental region (Sluys et al. 1998), while only three species have been recorded from China, viz., *D. japonica* Ichikawa & Kawakatsu, 1964, *D. sinensis* Chen & Wang, 2015, and *D. umbonata* Song & Wang, 2020 (Kawakatsu et al. 1976; Chen et al. 2015; Song et al. 2020). Hainan Island is the largest tropical island of China and forms part of the Indo-Burma biodiversity hotspot (Mittermeier et al. 2005, 2011) and represents an endemic bird area (Stattersfield et al. 1998). Although Liu (1993, map on p. 158) recorded the occurrence of *Dugesia* on Hainan Island from three localities, no information was provided on the taxonomic identity of the species. Therefore, in this paper we describe for the first time two new species of *Dugesia* from Hainan Island, which also form new species to the planarian fauna of China, on the basis of an integrative taxonomic approach, using morphological, karyological, and molecular data.

## Materials and methods

### Specimen collection and culturing

**Figure 1.**
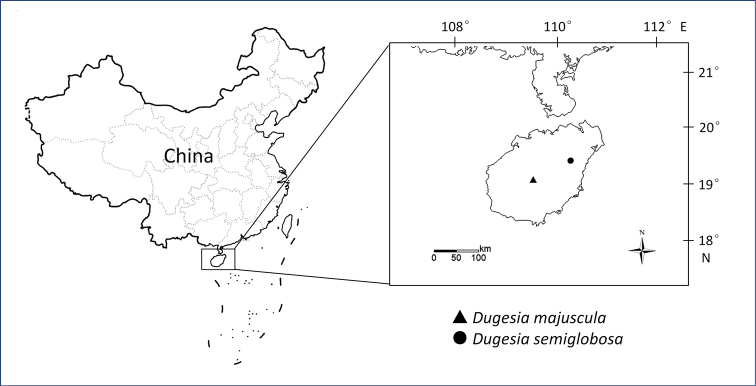
Collection sites of *Dugesia* on Hainan Island.

Specimens were collected during 2016–2018 from under stones in springs with the help of a paint brush (for sampling localities, see Fig. 1). The worms were transferred to plastic bottles filled with spring water, which were placed in a cooler, containing an ice bag, during transportation to the laboratory. In the laboratory, the planarians were cultured in autoclaved tap water at 20 °C and fed with fresh beef liver once per week. The worms were starved for at least one week before being used in karyotype studies, histological studies, or DNA extraction. Images of the external appearance of the worms were obtained by using a digital camera attached to a stereo-microscope.

### Histology and karyology

Histological sections were prepared as described by Dong et al. (2017). In brief, worms were killed with 1% nitric acid and thereafter fixed in Bouin’s fluid for ~ 24 h, then rinsed in 70% ethanol, and, subsequently, dehydrated in an ascending series of ethanol solutions, after which the animals were cleared in xylene and embedded in synthetic paraffin. Serial sections were made at intervals of 6–8 μm and were stained with haematoxylin-eosin. Images were acquired by a digital camera attached to a compound microscope. Preparations of specimens have been deposited in the Zoological Museum of the College of Life Science of Henan Normal University (**ZMHNU**), Xinxiang, China.

Karyological preparations were made by means of the air-drying technique, following Dong et al. (2017). Well-spread sets of metaphase plates from five randomly selected individuals were used for karyotype analysis; karyotype parameter measurements were carried out as described by Chen et al. (2008). Chromosomal nomenclature follows Levan et al. (1964).

### DNA extraction, amplification, sequencing, and phylogenetic analysis

Total genomic DNA was extracted by using the QIAamp DNA Mini Tissue Kit (Qiagen, Germany), according to the manufacturer’s protocols. Fragments of the Cytochrome C oxidase subunit I (COI) and internal transcribed spacer-1 (ITS-1) were amplified using specific primers (see Suppl. material 2: Table S1 for sequences and annealing temperatures). Premix Ex TaqHot Start Version (TaKaRa, Otsu Japan) was used for the polymerase chain reaction (PCR). Amplifications were conducted in a final volume of 30 µL under the following conditions: 5 min at 94 °C, 35 cycles of 40 s at 94 °C, an annealing step for 30 s, and 1min at 72 °C, and 5 min at 72 °C as a final extension (for annealing temperatures see Suppl. material 2: Table S1). Purification of PCR products and sequencing were done by GENEWIZ (Tianjin, China). Sequencing reactions were performed with the same primers used to amplify the fragments. All specimens were sequenced for both forward and reverse DNA strands. Chromatograms were visually checked. In the case of *Dugesia majuscula* Chen & Dong, sp. nov., we were able to extract DNA from no less than 12 specimens. With respect to *D. semiglobosa* Chen & Dong, sp. nov., we could only extract DNA from four specimens. For both of the two new species COI and ITS-1 were amplified.

In order to determine whether the presumed new species are molecularly different from other *Dugesia* species, we performed a phylogenetic analysis. In the ingroup we included the two new species as well as 28 other *Dugesia* species from the Oriental, Australasian, Mediterranean, Eastern and Western Palearctic regions; the freshwater species *Schmidtea mediterranea* Benazzi et al., 1975 was chosen as outgroup taxon, since this genus forms the sister-group of the genus *Dugesia* (Álvarez-Presas et al. 2008; for GenBank accession numbers, see Table 1).

**Table 1.** GenBank accession numbers of COI and ITS-1 sequences used for molecular analysis.

Species	GenBank		Species	GenBank	
	COI	ITS-1		COI	ITS-1
*D. aethiopica*	KY498845	KY498785	*D. improvisa*	KC006987	KC007065
*D. afromontana*	KY498846	KY498786	*D. japonica*	FJ646990	FJ646904
*D. arcadia*	KC006971	KC007044	*D. liguriensis*	FJ646992	FJ646907
*D. ariadnae*	KC006972	KC007048	*D. majuscula*	MW533425	MW533591
*D. batuensis*	KF907818	KF907815	*D. malickyi*	KC006990	KC007069
*D. benazzii*	FJ646977 + FJ646933	FJ646890	*D. naiadis*	KF308756	
*D. bengalensis*		FJ646897	*D. notogaea*	FJ646993 + FJ646945	FJ646908
*D. bifida*	KY498851	KY498791	*D. ryukyuensis*	AF178311	FJ646910
*D. cretica*	KC006976	KC007050	*D. semiglobosa*	MW525210	MW526992
*D. damoae*	KC006979	KC007057	*D. sicula*	FJ646994+FJ646947	DSU84356
*D. deharvengi*	KF907820	KF907817	*D. sigmoides*	KY498849	KY498789
*D. effusa*	KC006983	KC007058	*D. sinensis*	KP401592	
*D. elegans*	KC006984	KC007063	*D. subtentaculata*	FJ646995 + FJ646949	DSU84369
*D. gibberosa*	KY498857	KY498803	*D. umbonata*	MT176641	MT177211
*D. gonocephala*	FJ646986 + FJ646941	FJ646901	*S. mediterranea*	JF837062	AF047854
*D. hepta*	FJ646988 + FJ646943	FJ646902			

Nuclear ribosomal markers were aligned online with MAFFT (Online Version 7.247) using the G-INS-i algorithm (Katoh and Standley 2013), and checked by using BioEdit v7.2.6.1. For alignments of the protein-coding COI sequences we used the TranslatorX pipeline (http://translatorx.co.uk; Abascal et al. 2010). Nucleotide sequences were translated into amino acid sequences (Translation table 9), followed by MAFFT, using the FFT-NS-2 progressive alignment method, and checked by using BioEdit v7.2.6.1, and then back-translated to nucleotide sequences. Since automated removal of gap columns and variable regions had been reported to negatively affect the accuracy of the inferred phylogeny (Dessimoz and Gil 2010; Tan et al. 2015), the Gblocks option (Talavera and Castresana 2007) was disabled. The concatenated sequences for the phylogenetic analysis were in the order ITS-1+ COI and consisted of a total of 1473 bp, including 4.4% missing data. In the concatenated sequences, missing data had been coded as “?”.

MrBayes v3.2 (Ronquist et al. 2012) and RaxML v8.2.10 (Stamatakis 2014) were used to infer phylogenies with the Bayesian Inference (BI) and Maximum-likelihood (ML) methods, respectively. For BI a run of 3 million generations and 25% burn-in was used under the GTR+I+G model. For the ML analysis, 10,000 replicates were performed under the GTR+I+G model. BI and ML trees were visualized and edited using Figtree v1.4.3.

Genetic distances were calculated for CO1 and ITS-1 with the help of MEGA 6.06 (Tamura et al. 2013) under the Kimura 2-parameter substitution model (Lázaro et al. 2009; Solà et al. 2013).

## Results

### Molecular phylogeny

We obtained fragments of sequences of COI and ITS-1 from 12 specimens of *D. majuscula* and four specimens of *D. semiglobosa*. The final alignments of these fragments of the nuclear ribosomal internal spacer ITS-1 and the mitochondrial gene COI were 676 and 816 base pairs (bp), respectively. In the two populations of *D. majuscula* and *D. semiglobosa*, there was no variation in COI and ITS-1.

**Figure 2.**
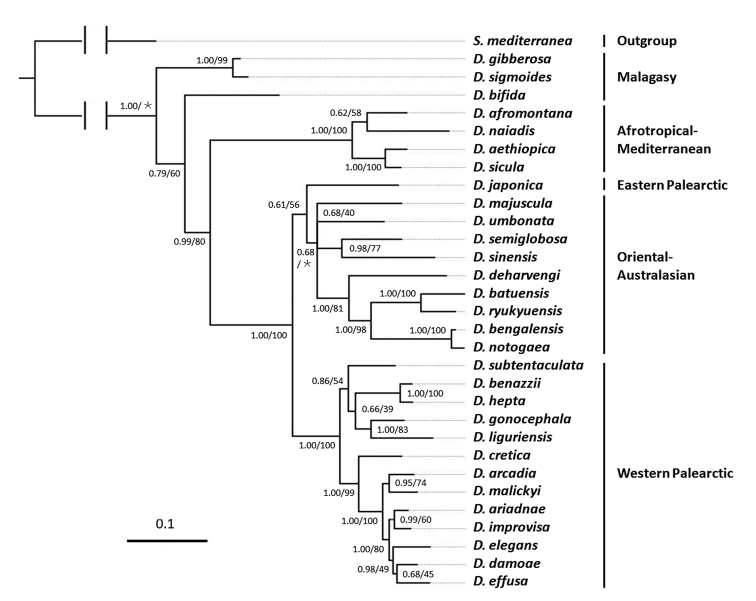
Phylogenetic tree obtained from Bayesian analysis of the concatenated dataset. Numbers at nodes indicate support values (posterior probability/bootstrap). ∗: Bootstrap value not applicable to the node, because of different topologies of trees obtained by BI and ML methods. Scale bar: substitutions per nucleotide position.

The phylogenetic trees and their supporting values resulting from the analysis of the concatenated dataset are very similar for both ML and BI, differing only in nodes weakly supported in at least one of the methodologies (Fig. 2; Suppl. material 1: Figure S1).The new species *D. majuscula* and *D. semiglobosa* occupy separate branches that are clearly differentiated from their congeners. Interestingly, these two species from Hainan Island are not each other’s closest relatives, as *D. semiglobosa* shares a sister-group relationship with *D. sinensis*, forming a clade that is part of a polytomy that comprises also three other branches, viz. *D. majuscula*, *D. umbonata*, and a clade comprising five species from the Oriental and Australasian regions (*D. deharvengi* Kawakatsu & Mitchell, 1989, *D. batuensis* Ball, 1970, *D. ryukyuensis* Kawakatsu, 1976, *D. bengalensis* Kawakatsu, 1983, *D. notogaea* Sluys & Kawakatsu, 1998).

In addition to the fact that the new species occupy separate branches in the phylogenetic tree, separate species status of *D. majuscula* and *D. semiglobosa* is supported also by our analysis of the genetic distances among the species included in our study, albeit that both COI and ITS-1 distances vary greatly among species (Suppl. material S2, S3). With respect to COI, the highest distance value between *D. majuscula* and its Oriental-Australasian congeners is 22.02%, while the lowest distance value is 13.68%. The highest distance value between *D. semiglobosa* and the Oriental-Australasian congeners is 21.06%, while the lowest distance value is 10.61%. Furthermore, there is a 19.52% difference between the two new species.

With respect to ITS-1, the highest distance value between *D. majuscula* and its Oriental-Australasian congeners is 12.52%, while the lowest distance value is 5.82%. The highest distance value between *D. semiglobosa* and its Oriental-Australasian congeners is 15.74%, while the lowest distance value is 9.74%. For this marker the distance between the two new species is 5.83% (Suppl. material 4: Table S3).

### Systematic account

**Order Tricladida Lang, 1884**

**Suborder Continenticola Carranza, Littlewood, Clough, Ruiz-Trillo, Baguñà & Riutort, 1998**

**Family Dugesiidae Ball, 1974**

**Genus *Dugesia* Girard, 1850**

#### Dugesia semiglobosa

Taxon classificationAnimaliaAsteralesAsteraceae

Chen & Dong sp. nov.

AAB66DF2-7FFD-5423-AEB5-ECDD3AB77E10

http://zoobank.org/59911F90-F4AC-4F86-8D54-9551D8ED67DF

**Figure 3.**
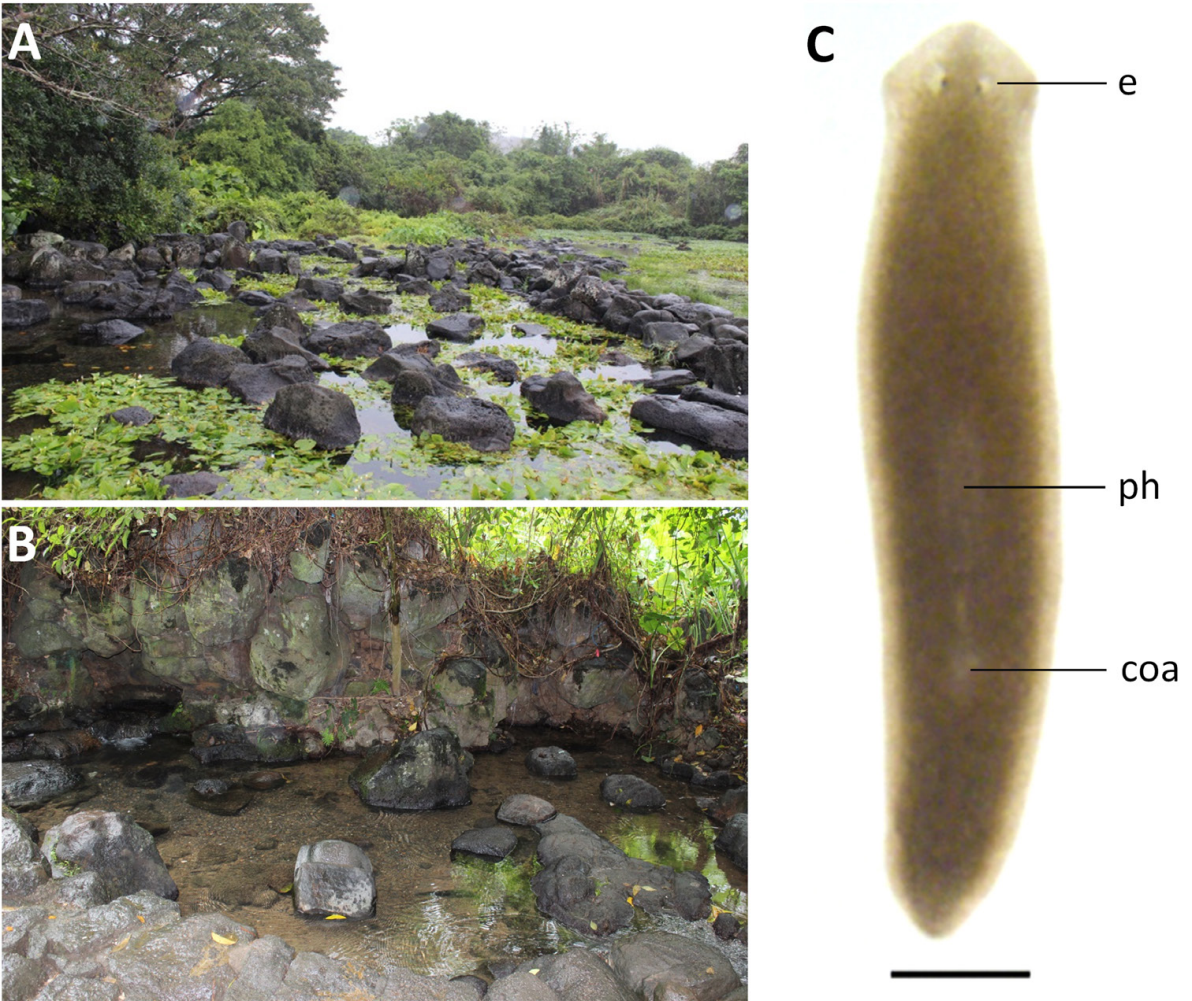
Habitat and external appearance of *Dugesia semiglobosa***A** sampling site **B** habitat **C** sexually mature, living individual (anterior is to the up). Abbreviations: coa: copulatory apparatus; e: eye; ph: pharynx. Scale bar: 1 mm.

**Figure 4.**
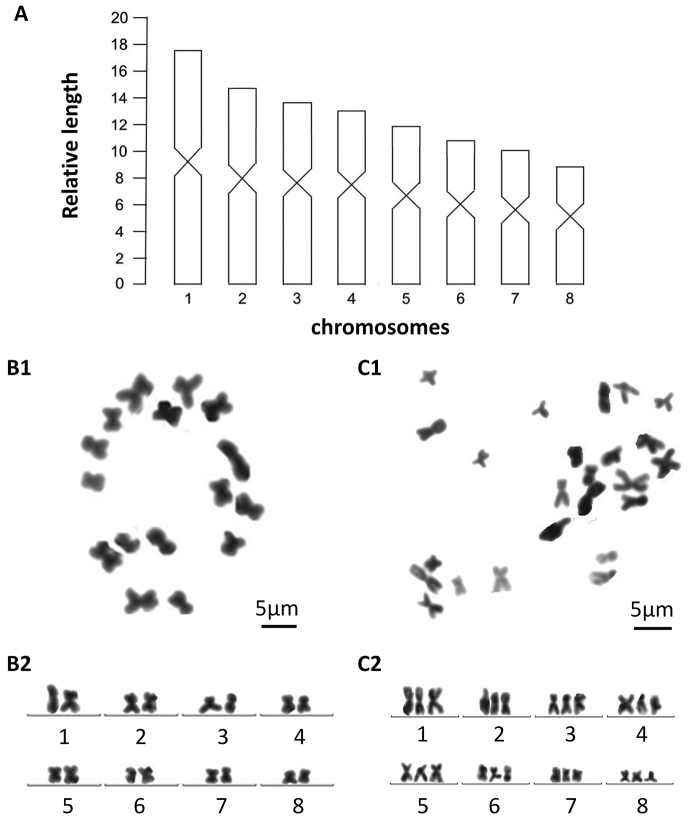
*Dugesia semiglobosa***A** idiogram **B1** metaphasic plate of diploid cell **B2** karyogram of diploid cell **C1** metaphasic plate of triploid cell **C2** karyogram of triploid cell.

**Figure 5.**
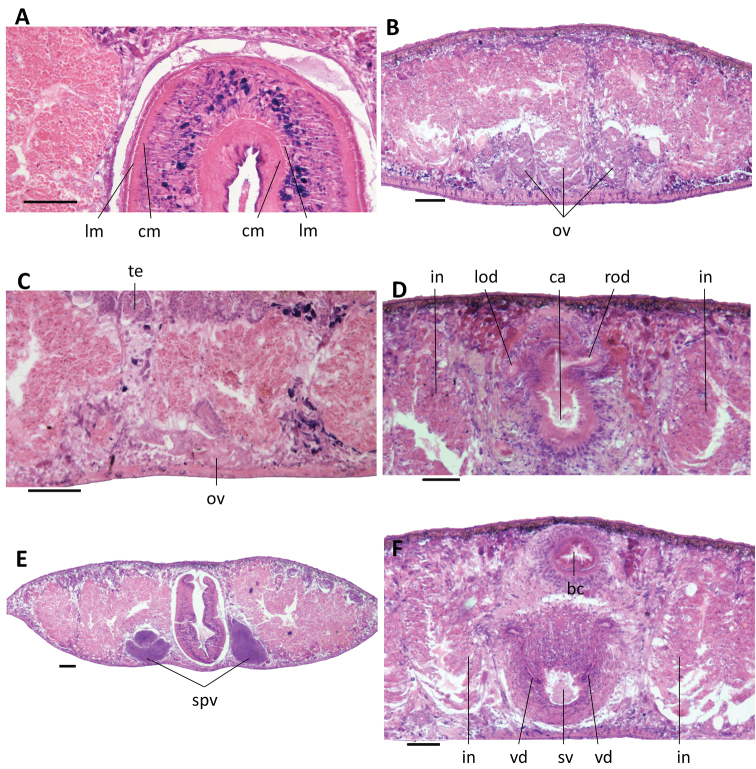
*Dugesia semiglobosa***A** transverse section of paratype JWT 6, showing longitudinal and circular muscles in outer and inner pharyngeal musculature **B** transverse section of paratype JWT 7, showing hyperplasic ovaries. **C** sagittal section of holotype JWT 5, showing testes and small ovary **D** transverse section of paratype JWT 7, showing symmetrical openings of the oviducts **E** transverse section of paratype JWT 6, showing spermiducal vesicles **F** transverse section of paratype JWT 7, showing openings of the vasa deferentia into the seminal vesicle. The anterior is to the front in **A, B, D, E, F**, and anterior is to the right in **C**. Abbreviations: bc: bursal canal; ca: common atrium; cm: circular muscles; in: intestine; lm: longitudinal muscles; lod: left oviduct; ov: ovary; rod: right oviduct; spv: spermiducal vesicle; sv: seminal vesicle; te: testis; vd: vas deferens. Scale bars: 100 μm.

**Figure 6.**
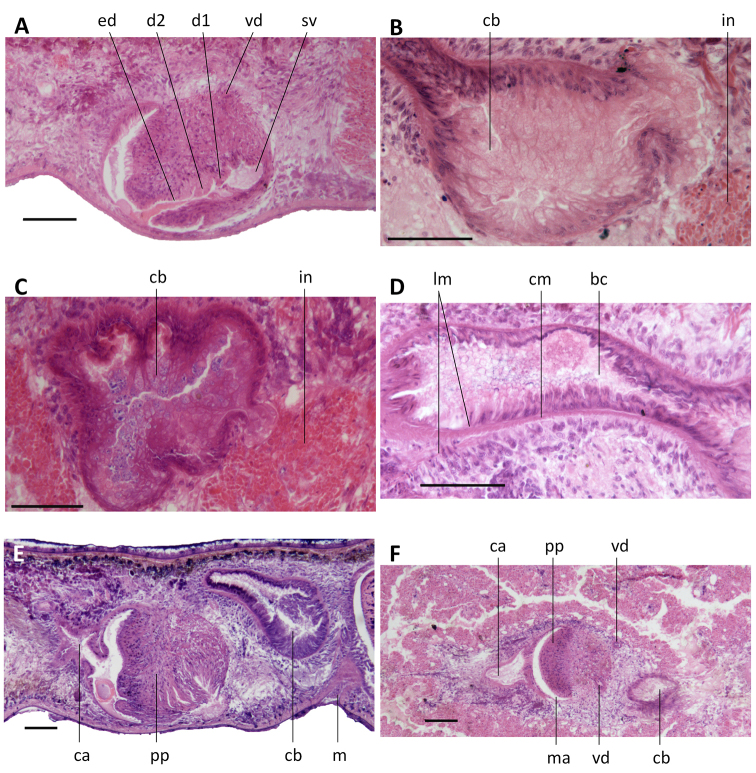
*Dugesia semiglobosa***A** sagittal section of holotype JWT 5, showing reniform seminal vesicle, two diaphragms in the ejaculatory duct, ventral course of the ejaculatory duct, and hemispherical penis papilla **B** sagittal section of holotype JWT 5, showing copulatory bursa. **C** transverse section of paratype JWT 7, showing copulatory bursa **D** sagittal section of holotype JWT 5, showing bursal canal and its surrounding musculature **E** sagittal section of holotype JWT 9, showing small copulatory bursa, and mouth **F** horizontal section of paratype JWT 4, showing hemispherical penis papilla. The anterior is to the right in **A, B, D, E, F** and anterior is to the front in **C**. Abbreviations: bc: bursal canal; ca: common atrium; cb: copulatory bursa; cm: circular muscles; d: diaphragm; ed: ejaculatory duct; in: intestine; lm: longitudinal muscles; m: mouth; ma: male atrium; pp: penis papilla; sv: seminal vesicle; vd: vas deferens. Scale bars: 100 μm.

**Figure 7.**
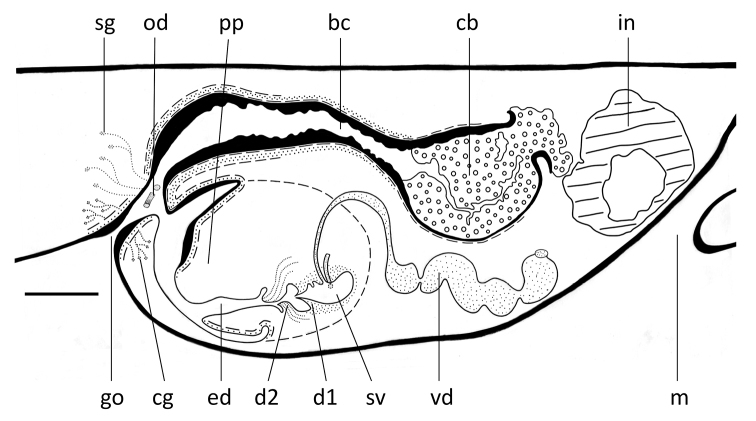
*Dugesia semiglobosa*. Sagittal reconstruction of the copulatory apparatus of the holotype (anterior is to the right). Abbreviations: bc: bursal canal; cb: copulatory bursa; cg: cement glands; d: diaphragm; ed: ejaculatory duct; go: gonopore; in: intestine; m: mouth; od: oviduct; pp: penis papilla; sg: shell glangs; sv: seminal vesicle; te: testis; vd: vas deferens. Scale bar: 100 μm.

Figures 3 , 4 , 5 , 6 , 7

##### Material examined.

***Holotype***: ZMHNU-JWT5, Jiuwentang village (19°23'10"N, 110°19'42"E; alt. 80 m above sea level (a.s.l.), Anding County, Hainan Province, China, 24 February 2018, coll. GW Chen and co-workers, sagittal sections on 17 slides.

***Paratypes***: ZMHNU-JWT1, ibid., sagittal sections on 17 slides; ZMHNU-JWT2, ibid., sagittal sections on 14 slides; ZMHNU-JWT3, ibid., sagittal sections on 16 slides; ZMHNU-JWT4, ibid., horizontal sections on 7 slides; ZMHNU-JWT6, ibid., transverse sections on 33 slides; ZMHNU-JWT7, ibid., transverse sections on 30 slides; ZMHNU-JWT8, ibid., sagittal sections on 49 slides; ZMHNU-JWT9, ibid., sagittal sections on 30 slides.

##### Diagnosis.

*Dugesia semiglobosa* is characterized by the following features: hemispherical, asymmetrical penis papilla with ventrally displaced ejaculatory duct opening terminally at tip of penis papilla; absence of duct intercalated between seminal vesicle and diaphragm; vasa deferentia separately opening into mid-dorsal portion of intrabulbar seminal vesicle; two diaphragms in the ejaculatory duct; symmetrical openings of oviducts into bursal canal; copulatory bursa formed by expansion of bursal canal, lined with complex stratified epithelium, which projects through opening in bursa towards intestine, without having open communication with the gut; mixoploid chromosome complement diploid (2n = 16) and triploid (3n = 24); chromosomes metacentric.

##### Etymology.

The specific epithet is derived from the Latin *semis*, half, and *globosus*, spherical, and alludes to the hemispherical penis papilla.

##### Habitat and reproduction.

Specimens were collected from Jiuwentang volcano spring at an altitude of 80 m a.s.l. and with a water temperature of 23 °C. This spring is the third largest volcano spring in China, while it is also its largest selenium-rich spring (Fig. 3A, B). None of the animals was sexually mature at collection. However, after having been kept under laboratory conditions for ~ 150 days, the animals sexualized and laid cocoons. Newly laid cocoons are yellow, but turn dark brown after 2 to 3 days. Cocoons are spherical in shape (1 mm in diameter) and provided with a stalk. Thus far, none of the cocoons hatched, thus, most likely being infertile.

##### Karyology.

Each of the five, randomly selected specimens exhibited mixoploid chromosome complements. In a total of 100 metaphase plates examined, 67 exhibited diploid chromosome portraits of 2n = 2x = 16, while in 20 plates chromosome complements were triploid with 2n = 3x = 24 chromosomes (Fig. 4); chromosome complements of the remaining 13 plates could not be determined, due to either lack of well-dispersed chromosomes or over-dispersed sets of chromosomes. All chromosomes were metacentric; karyotype parameters, including relative length, arm ratio, and centromeric index, are given in Table 2. The first pair of chromosomes is clearly larger than others, being 1.8 times larger than the shortest chromosome. Chromosomal plates and idiogram are shown in Fig. 4.

**Table 2.** Karyotype parameters (mean values and standard deviations) of *Dugesia semiglobosa*; m: metacentric.

Chromosome	Relative length	Arm ratio	Centromeric index	Chromosome type
1	17.43 ± 0.93	1.12 ± 0.09	47.31 ± 1.83	m
2	14.71 ± 0.46	1.19 ± 0.09	45.88 ± 1.69	m
3	13.69 ± 0.45	1.30 ± 0.18	43.86 ± 3.53	m
4	12.93 ± 0.46	1.34 ± 0.14	43.21 ± 2.54	m
5	11.78 ± 0.26	1.29 ± 0.11	43.82 ± 2.12	m
6	10.80 ± 0.17	1.27 ± 0.06	44.33 ± 1.35	m
7	9.92 ± 0.53	1.28 ± 0.10	44.24 ± 1.97	m
8	8.72 ± 0.64	1.21 ± 0.05	45.40 ± 1.02	m

##### Description.

Body of living asexual specimens is 4–6 mm in length and 0.72–0.85 mm in width, while in sexualized animals the body is 8–12 mm in length and 1.25–1.51 mm in width. Two eyes located in the center of the head, being situated in pigment-free patches. Each pigmented eye cup houses numerous photoreceptor cells. Head of low triangular shape and provided with two blunt auricles. Body light brown dorsally, excepting the pale body margin and accumulations of pigment following the outline of the pharynx. Ventral surface is paler than the dorsal one (Fig. 3C).

Pharynx situated in the mid-region of the body, measuring ~ 1/5^th^ of the body length (Fig. 3C). Mouth opening located at the posterior end of the pharyngeal pocket. Outer pharyngeal musculature composed of a subepidermal layer of longitudinal muscles, followed by a thick layer of circular muscles; extra inner layer of longitudinal muscles is absent (Fig. 5A). The inner pharyngeal musculature consists of a subepithelial layer of circular muscle, followed by a layer of longitudinal muscle, the former being thicker than the latter (Fig. 5A).

In specimen JWT-7 the ventrally placed ovaries are clearly hyperplasic and fused to form a single mass that extends into the lateral regions of the body (Fig. 5B). In other specimens examined (JWT-2, JWT-3, JWT-5, JWT-6, JWT-10), the gonads are generally atypical, in that they are very small (Fig. 5C). Only in specimen JWT-8 the ovaries are more or less of normal size. In general, ovaries are situated at a short distance behind the brain.

From the ovaries the oviducts run ventrally in a caudal direction to the level of the genital pore, after which they curve dorso-medially to open separately and symmetrically into the ventral portion of the bursal canal, close to its communication with the atrium (Figs 5D, 7).

The small, dorsally located testes are well developed and provided with mature spermatozoa (Fig. 5C). Testicular follicles are arranged on either side of the midline of the body in nine or ten longitudinal zones, extending from the posterior level of the ovaries to almost the posterior end of the body. The vasa deferentia, filled with spermatozoa, expand to form spermiducal vesicles at the level of the pharynx that occupy < 1/3^rd^ of the dorso-ventral space (Fig. 5E). At the level of the penis bulb the vasa deferentia decrease in diameter and bend sharply towards the dorsal body surface and upon recurving ventrad they penetrate the dorso-lateral wall of the penis bulb to open separately and symmetrically into the mid-dorsal portion of the seminal vesicle (Figs 5F, 6A, 7). The sperm ducts are lined with nucleated cells and are surrounded by a layer of circular muscles. The reniform seminal vesicle is lined by a flat, nucleated epithelium and is surrounded by intermingled muscle fibers (Figs 5F, 6A,). The seminal vesicle opens into the ejaculatory duct via a small, pointed diaphragm. A second, rather large and blunt diaphragm is located in the proximal portion of the ejaculatory duct (Figs 6A, 7). The ejaculatory duct is lined by an infranucleated epithelium; we were unable to discern any musculature around the duct. The ejaculatory duct follows a noncentral, ventrally displaced course through the penis papilla, opening at its tip, thus resulting in an asymmetrical penis papilla in which the dorsal lip is much larger than the ventral one (Figs 6A, 7).

The complete penis, comprising papilla and bulb, is nearly spherical, with the penis papilla being a hemispherical structure that is covered with an infranucleated epithelium, which is underlain by a subepithelial layer of circular muscle, followed by a layer of longitudinal muscle fibres (Fig. 6A). The penis papilla almost completely occupies the male atrium, the latter communicating with the common atrium via a slight constriction (Figs 6E, F, 7). The common atrium opens to the exterior via a gonoduct, which is lined by a columnar epithelium and receives the openings of abundant cement glands.

From its point of communication with the common atrium, the bursal canal gradually expands in diameter, meanwhile curving anteriad, while running on the left side of the male copulatory apparatus (Figs 6D, 7). The bursal canal is lined with columnar, nucleated, ciliated cells and is surrounded by a subepithelial layer of longitudinal muscles, followed by a layer of circular muscle (Figs 6D, 7). An ectal reinforcement layer of longitudinal muscles runs from the vaginal region to approximately halfway along the bursal canal. Shell glands discharge their erythrophil secretion into the vaginal region of the bursal canal, near the oviducal openings.

More or less dorsally to the penis bulb, the bursal canal first decreases somewhat in diameter but thereafter greatly expands to give rise to a more or less globular structure immediately in front of the male complex. This globular structure may be called a copulatory bursa since it occupies the same position as in other species of *Dugesia*, or freshwater planarians in general. The bursa is lined with a complex type of stratified epithelium. The basal portion of this epithelium consists of more or less cuboidal, nucleated cells and is basically a continuation of the lining epithelium of the rest of the bursal canal, albeit that there the cells are columnar. This basal layer is followed by a thick zone of stratified, non-nucleated squamous epithelium, leaving very little room for any lumen within the bursa. The cells of this squamous layer have an irregular shape, while those in the top zone, near the lumen, are vacuolated and provided with granular, cyanophil inclusions. The bursa is surrounded by a layer of longitudinal muscles (Figs 6C, 7). However, at one point this muscle layer is interrupted because of the presence of an opening in the bursa. This opening is located, more or less, at the antero-dorsal wall of the bursa. A portion of the squamous inner lining of the bursa projects through the opening and approaches and/or touches portions of the gut that are in its proximity. However, in none of the specimens examined we discerned an open connection between bursa and intestine.

##### Discussion.

The curious copulatory bursa of *D. semiglobosa* is unparalleled among species of *Dugesia*, or freshwater planarians in general. Generally, copulatory bursae are lined with an epithelium consisting of tall columnar, vacuolated, and nucleated cells, while they are surrounded by only a very weak musculature. The structure of the bursa of *D. semiglobosa* differs considerably from this ground-plan condition, as it is basically an expanded continuation of the bursal canal, albeit with a simpler coat of muscles and a more complex lining epithelium.

*Dugesia semiglobosa* exhibits a combination of three characteristic features (ventrally displaced ejaculatory duct, absence of duct intercalated between seminal vesicle and diaphragm, terminal opening of ejaculatory duct) that is found in only nine congeners, viz., *D. annandalei* Kaburaki, 1918, *D. damoae* De Vries, 1984, *D. didiaphragma* De Vries, 1988, *D. elegans* De Vries, 1984, *D. gibberosa* Stocchino & Sluys, 2017, *D. maghrebiana*Stocchino et al. 2009, *D. malickyi* De Vries, 1984, *D. naiadis* Sluys, 2013, and *D. sinensis.* Among these nine species, *D. semiglobosa* most closely resembles *D. didiaphragma* and *D. maghrebiana* in that these species also possess two diaphragms, in contrast to all other species mentioned. Presence of two diaphragms is a rare condition among species of *Dugesia* and is only known from three other species, viz., *D. bijuga* Harrath & Sluys, 2019, *D. machadoi* de Beauchamp, 1952, and *D. mirabilis* De Vries, 1988. However, in these three last-mentioned species the ejaculatory duct runs a central course through the penis papilla, in contrast to the ventral trajectory in *D. semiglobosa*. Further, there are ample other features that preclude assignment of our specimens to either of these Afrotropical species. Neither is it possible to assign our animals to *D. didiaphragma* or *D. maghrebiana* as they lack the large seminal vesicle enclosed by a highly muscularized, elongated penis bulb of the former and the knob-like extension on the penis papilla of the latter.

It is interesting to note that in all species in possession of two diaphragms, the small proximal diaphragm basically is formed by a non-glandular constriction of the seminal vesicle, while the true diaphragm is a larger structure and receives the secretion of penial glands, as usual for the diaphragm of species of *Dugesia*. The same situation applies to the two diaphragms in *D. semiglobosa*. It is noteworthy that in *D. mirabilis* both the proximal and distal diaphragm are glandular (De Vries 1988).

#### Dugesia majuscula

Taxon classificationAnimaliaAsteralesAsteraceae

Chen & Dong sp. nov.

4289D2EC-67C6-5D90-AE9C-83615FC11CE8

http://zoobank.org/C4E72273-DA7E-48DE-BFFC-B0F88DE47626

**Figure 8.**
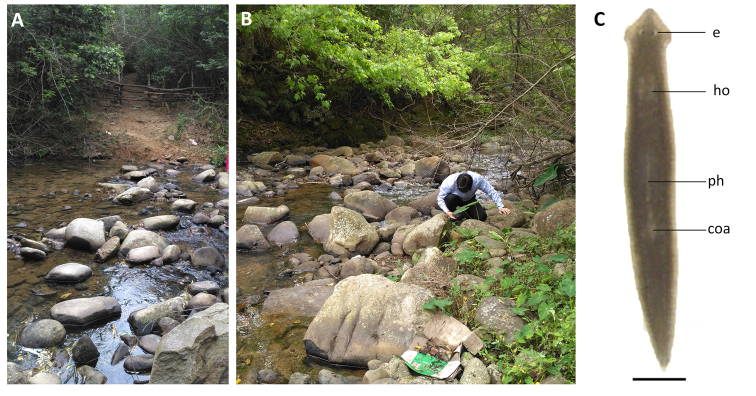
Habitat and external appearance of *Dugesia majuscula***A** sampling locality **B** habitat **C** sexually mature, living individual (anterior is to the up). Abbreviations: coa: copulatory apparatus; e: eye; ph: pharynx. Scale bar: 1 mm.

**Figure 9.**
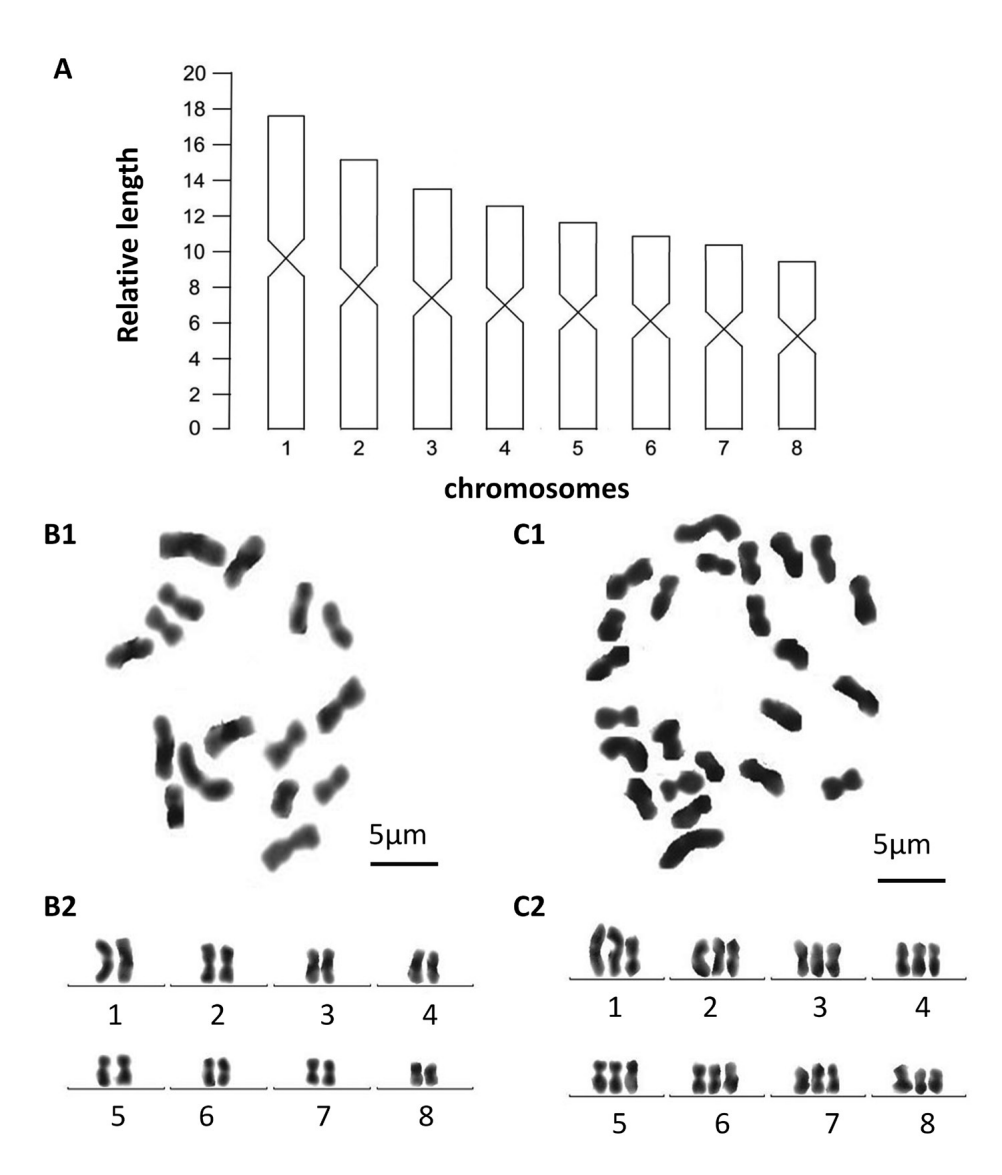
*Dugesia majuscula***A** idiogram **B1** metaphasic plate of diploid cell **B2** karyogram of diploid cell **C1** metaphasic plate of triploid cell **C2** karyogram of triploid cell.

**Figure 10.**
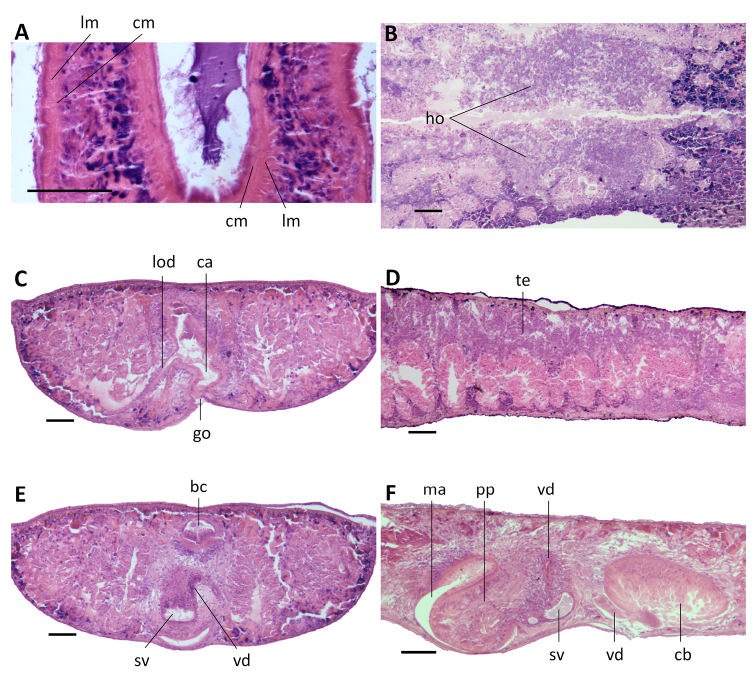
*Dugesia majuscula***A** transverse section of paratype YHYQ8, showing longitudinal and circular muscles in outer and inner pharyngeal musculature **B** horizontal section of paratype YHYQ 7, showing hyperplasic ovaries **C** transverse section of paratype YHYQ8, showing opening of the left oviduct **D** sagittal section of paratype YHYQ10, showing dorsal testes **E** transverse section of paratype YHYQ8, showing course of a vas deferens **F** sagittal section of paratype YHYQ3, showing opening of a vas deferens into seminal vesicle. The anterior is to the front in **A, C, E** and anterior is to the right in **B, D, F**. Abbreviations: bc: bursal canal; ca: common atrium; cb: copulatory bursa; cm: circular muscles; go: gonopore; ho: hyperplasic ovaries; lm: longitudinal muscles; lod: left oviduct; ma: male atrium; pp: penis papilla; sv: seminal vesicle; te: testis; vd: vas deferens. Scale bars: 100 μm.

**Figure 11.**
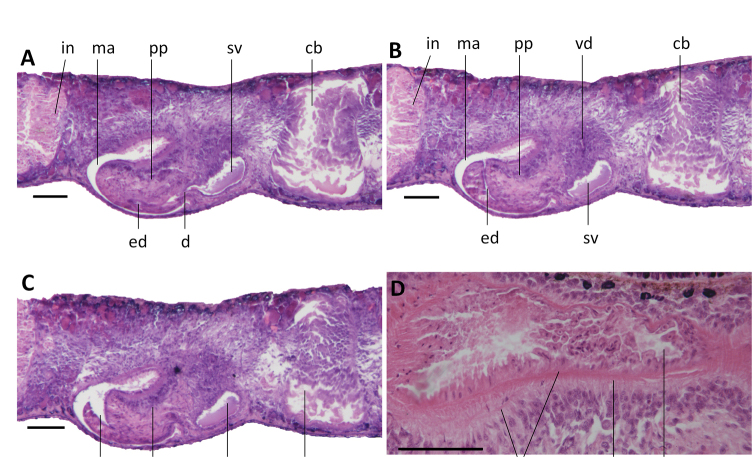
*Dugesia majuscula***A** sagittal section of holotype YHYQ4, showing seminal vesicle, ejaculatory duct, small diaphragm, penis papilla, and copulatory bursa **B** Sagittal section of holotype YHYQ4, showing seminal vesicle, ejaculatory duct, penis papilla, and copulatory bursa **C** sagittal section of paratype YHYQ5, showing terminal and dorsal opening of ejaculatory duct **D** sagittal section of paratype YHYQ9, showing bursal canal and its musculature. The anterior is to the right in **A, B, C, D**. Abbreviations: bc: bursal canal; cb: copulatory bursa; cm: circular muscles; d: diaphragm; ed: ejaculatory duct; in: intestine; lm: longitudinal muscles; ma: male atrium; pp: penis papilla; sv: seminal vesicle; vd: vas deferens. Scale bars: 100 μm.

**Figure 12.**
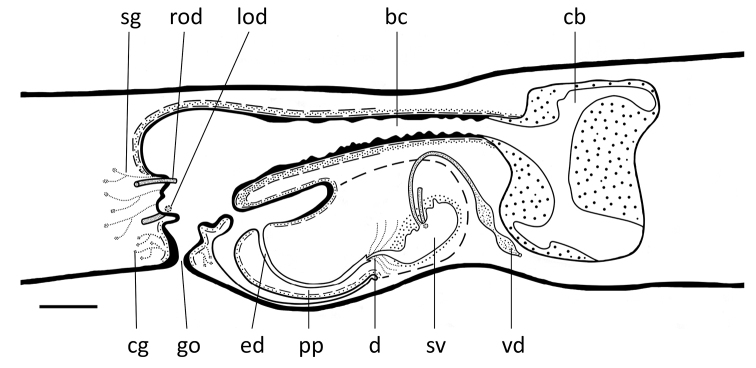
*Dugesia majuscula.* Sagittal reconstruction of the copulatory apparatus of the holotype (anterior is to the right). Abbreviations: bc: bursal canal; cb: copulatory bursa; cg: cement glands; d: diaphragm; ed: ejaculatory duct; go: gonopore; lod: left oviduct; pp: penis papilla; rod: right oviduct; sg: shell glangs; sv: seminal vesicle; vd: vas deferens. Scale bar: 100 μm.

Figures 8 , 9 , 10 , 11 , 12

##### Material examined.

***Holotype***: ZMHNU-YHYQ 4, sagittal sections on 36 slides, Yingge mountain, Qiongzhong County, Hainan Province, China (19°3'24"N, 109°33'49"E), 24 February 2018, altitude 584 m a.s.l., coll. GW Chen and co-workers.

***Paratypes***: ZMHNU-YHYQ1, ibid. sagittal sections on 45 slides; ZMHNU-YHYQ2, ibid., sagittal sections on 18 slides; ZMHNU-YHYQ3, ibid., sagittal sections on 24 slides; ZMHNU-YHYQ5, ibid., sagittal sections on 32 slides; ZMHNU-YHYQ 6, horizontal sections on 18 slides; ZMHNU-YHYQ 7, horizontal sections on12 slides; ZMHNU-YHYQ8, ibid., transverse sections on 24 slides; ZMHNU-YHYQ9, ibid., sagittal sections on 15 slides; ZMHNU-YHYQ10, ibid., sagittal sections on 19 slides.

##### Diagnosis.

*Dugesia majuscula* is characterized by the following features: oviducts opening asymmetrically into female reproductive system; hyperplasic ovaries; expanded posterior section of bursal canal; vasa deferentia separately opening into mid-dorsal portion of seminal vesicle; asymmetrical penis papilla due to ventral course of ejaculatory duct, which has subterminal and dorsal opening at tip papilla; mixoploid chromosome complement diploid (2n=16) and triploid (3n = 24), with metacentric chromosomes.

##### Etymology.

The specific epithet is derived from the Latin adjective *majusculus*, somewhat larger, and alludes to expanded portion of the bursal canal.

##### Habitat and reproduction.

Animals were collected from a freshwater stream in the Yingge Mountains, at a water temperature of 19 °C and an altitude of 584 m a.s.l. (Fig. 8A, B). Approximately 40 individuals were collected in February 2018, none of which was sexually mature. During the first period of 80–90 days (March to May) in the laboratory culture, worms only showed asexual reproduction by means of fission. However, during the following period of 40–60 days, seven individuals sexualized, while after another 180 days, 1/3 of the animals sexualised, although thus far they have not produced any cocoons.

##### Karyology.

Each of the five, randomly selected specimens exhibited mixoploid chromosome complements. In a total of 100 metaphase plates examined, 75 exhibited diploid chromosome complements of 2n = 2x = 16, while in 18 plates chromosome complements were triploid with 2n = 3x = 24 chromosomes (Fig. 9); chromosome complements of the remaining seven plates could not be determined, due to either lack of well-dispersed chromosomes or over-dispersed sets of chromosomes. All chromosomes were metacentric; karyotype parameters, including relative length, arm ratio, and centromeric index, are given in Table 3. The first pair of chromosomes is clearly larger than the others, being 1.86 times larger than the shortest chromosome. Chromosomal plates and idiogram are shown in Fig. 9.

**Table 3.** Karyotype parameters (mean values and standard deviations) of *Dugesia majuscula*; m: metacentric.

Chromosome	Relative length	Arm ratio	Centromeric index	Chromosome type
1	17.76 ± 0.82	1.20 ± 0.08	45.66 ± 1.69	m
2	14.95 ± 0.72	1.15 ± 0.08	46.61 ± 1.67	m
3	13.30 ± 0.33	1.19 ± 0.04	45.70 ± 0.74	m
4	12.27 ± 0.31	1.25 ± 0.09	44.68 ± 1.81	m
5	11.47 ± 0.46	1.31 ± 0.17	43.58 ± 3.37	m
6	10.77 ± 0.44	1.19 ± 0.10	46.06 ± 2.07	m
7	10.21 ± 0.57	1.20 ± 0.08	45.77 ± 1.50	m
8	9.28 ± 0.34	1.25 ± 0.17	44.73 ± 3.13	m

##### Description.

Body size of living asexual specimens is 5–8 mm in length and 0.65–0.78 mm in width, while in sexualized specimens the body measures 10–16 mm in length and 1.05–1.36 mm in width. Head of low triangular shape, provided with two blunt auricles and two eyes located in pigment-free patches (Fig. 8C). Each pigmented eye cup is provided with numerous retinal cells. Dorsal surface brown with numerous dark spots (Fig. 8C); ventral surface paler than dorsal one and provided with only scattered small, dark spots.

Pharynx positioned in the mid-region of the body and measuring ~ 1/6^th^ of the body length (Fig. 8C); the mouth opening is situated at the hind end of the pharyngeal pocket. The pharyngeal outer musculature is composed of an outer, subepithelial layer of longitudinal muscles, followed by a layer of circular muscles. An extra longitudinal muscle layer internally to the circular muscles is absent (Fig. 10A). Inner pharyngeal musculature composed of an outer layer of longitudinal muscles and a subepithelial layer of circular muscles, the latter being thicker than the outer layer (Fig. 10A).

Ovaries hyperplasic, with several scattered masses at a short distance behind the brain, extending backwards over a distance of at least 800 μm (Fig. 10B) and occupying the entire dorso-ventral space. In live animals, the hyperplasic ovaries are visible through the dorsal body surface as two short, transparent stripes (Fig. 8C). From the ovaries, the oviducts run ventrally in a caudal direction to the level of the genital pore where they turn dorso-medially to open separately and asymmetrically into the female reproductive system. In point of fact, the right oviduct opens either into the most postero-ventral part of the expanded portion of the bursal canal or somewhat more ventrally (YHYQ-5), while the left oviduct opens into the common atrium, a distinct female atrium being absent (Figs 10C, 12).

The well-developed testes are situated dorsally and extend from the level of the ovaries to the posterior end of the body (Fig. 10D). Upon approaching the level of the penis bulb, composed of intermingled muscle fibers, the vasa deferentia curve dorso-mediad and near the postero-dorsal wall of the penis bulb the sperm ducts abruptly bend ventrad to penetrate the wall of the penis bulb. Within the bulb, the sperm ducts open separately and symmetrically into the mid-dorsal portion of a large reniform seminal vesicle (Figs 10E, F, 12). The sperm ducts are lined with nucleated cells and are surrounded by a layer of circular muscles. The seminal vesicle opens into the ejaculatory duct via a very small diaphragm positioned at a level that corresponds with the root of the penis papilla (Figs 11A, 12). The ejaculatory duct runs ventrally through the penial papilla and opens to the exterior by means of subterminal dorsal opening at the tip of the papilla (Figs 11A–C, 12). The ejaculatory duct is lined by a cuboidal epithelium; we were unable to discern any musculature around the ejaculatory duct.

The cylindrical penis papilla is covered by an epithelium that is underlain with a subepithelial layer of circular muscle, followed by a layer of longitudinal muscle fibers. As a result of the ventral course of the ejaculatory duct, the penis papilla is asymmetrical, with its dorsal lip being much larger than the ventral one. The basal part of the penis papilla has an oblique, ventro-caudal orientation, after which the penis papilla makes a bend, so that the rest of the papilla has a more or less horizontal orientation (Figs 11A–C, 12). The papilla almost completely occupies the male atrium, which communicates with the common atrium via a more or less pronounced constriction (Fig. 12). In turn, the latter communicates with a gonoduct, lined with a columnar epithelium and receiving the openings of abundant cement glands, which leads to the ventral gonopore.

The large sac-shaped copulatory bursa, which occupies the entire dorso-ventral space, is lined by a vacuolated epithelium provided with basal nuclei. There is hardly any musculature surrounding the bursa (Figs 11A–C, 12). From the postero-dorsal wall of the bursa, the bursal canal runs in a caudal direction to the left side of the male copulatory apparatus. Dorsally to the male atrium the bursal canal expands, particularly in dorso-ventral direction, to form a spacious posterior section that communicates with the common atrium. The bursal canal is lined with cylindrical, infranucleated, and ciliated cells and is surrounded by a subepithelial layer of longitudinal muscles, followed by a well-developed layer of circular muscle and a thin layer of longitudinal muscles. The outer longitudinal muscles constitute the ectal reinforcement layer, which extends from the vaginal region to approximately halfway along the bursal canal (Figs 11D, 12). Erythrophil shell glands open into the vaginal region of the bursal canal around the oviducal openings.

##### Discussion.

Our present task of comparing the features of *D. majuscula* with already known species of *Dugesia* is made considerably easier by the fact that it exhibits the unusual character of a subterminal dorsal opening of the ejaculatory duct at the tip of the penis papilla, which thus far has been reported for only one other species, viz., *D. umbonata* from southwest China (Song et al. 2020). In view of the sister-group relationship between these two species (Fig. 2), this subterminal, dorsal opening of the ejaculatory duct may be hypothesized to represent a synapomorphy for *D. umbonata* and *D. majuscula*.

Although *D. umbonata* exhibits also asymmetrical oviducal openings and a ventrally displaced ejaculatory duct, as in *D. majuscula*, it differs from the latter notably in the presence of a large, muscularized hump on its bursal canal, among other differences. A subterminal opening of the ejaculatory duct is not uncommon among species of *Dugesia*, but in most cases the openings are ventral, in contrast to the dorsal opening in *D. majuscula* and *D. umbonata*. The only exception is *D. hepta* Pala et al., 1981 but in this species the ejaculatory duct exits at the dorso-lateral tip of the penis papilla, while in this species the ejaculatory duct is dorsally displaced, in contrast to the ventral course in *D. majuscula* and *D. umbonata*. Furthermore, in *D. hepta* the oviducts open symmetrically into the bursal canal (Stocchino et al. 2005), whereas in *D. majuscula* and *D. umbonata* the oviducal openings are asymmetrical.

## General discussion

### Molecular phylogeny and distances

The topology of our phylogenetic tree (Fig. 2) basically accords with results from previous phylogenetic analyses (Lázaro et al. 2009; Solà et al. 2013; Stocchino et al. 2017; Song et al. 2020). Detailed comparison between these results and our tree are complicated since the various studies included different sets of species as well as different molecular markers, while it also serves little purpose as our sole aim was to assess the taxonomic status of the two new Chinese species. Song et al. (2020) already noted that because of the rather low number of *Dugesia* species for which molecular information is available, it is presently not well possible to analyse the historical biogeography of the genus.

For being able to interpret genetic distances between presumably new species in the delimitation of species boundaries, it is necessary to have well-supported information on distances between already well-known species. Presently, our information on genetic distances between species of *Dugesia* is rather limited. Harrath et al. (2019) found that for COI, the lowest distance value between species was 7%, while Stocchino et al. (2017) reported distances usually greater than 10% among species from Madagascar. Furthermore, Lázaro et al. (2009) and Solà et al. (2013) showed, respectively, that the lowest distance value between Mediterranean species generally is greater than 10% or 6%. With respect to the new species *D. majuscula* and *D. semiglobosa*, the lowest genetic distance value between these species and their Oriental-Australasian congeners was 13.68% and 14.26%, respectively, while there is a 19.52% difference between the two new species (Suppl. material 3: Table S2).The lowest distance values reported for ITS-1 were 6% between Malagasy species (Stocchino et al. 2017), and 7% and 1% for Mediterranean species (Lázaro et al. 2009; Solà et al. 2013). With respect to *D. majuscula* and *D. semiglobosa*, the lowest distance values between these species and other Oriental-Australasian congeners were 5.82% and 9.74%, respectively. For this marker, the distance between the two new species was 5.83% (Suppl. material 4: Table S3). Thus, these inter-species genetic distances for COI and ITS-1 support both the molecular phylogenetic and the anatomical results, which already indicated that *D. majuscula* and *D. semiglobosa* are well-separated from their congeners.

### Morphology

It is well-known that sexualized specimens from originally fissiparous populations generally develop hyperplasic ovaries (cf. Song et al. 2020 and references therein), excepting *D. benazzii* and *D. etrusca*, in which ex-fissiparous individuals did not develop hyperplasic ovaries (Stocchino et al. 2012; Stocchino and Manconi 2013). Furthermore, in such sexualized specimens with hyperplasic ovaries, testes may be under-developed or even be completely absent. However, such is not the case in *D. semiglobosa* and *D. majuscula*, as in both species the sexualized individuals possessed well-developed testes, while their vasa deferentia contained ample sperm, a condition that was previously observed also in *Dugesia bifida* Stocchino & Sluys, 2014 (Stocchino et al. 2014). Nevertheless, in the two new species cocoons were either not produced or were infertile.

It is noteworthy that in both new species from Hainan Island the vasa deferentia follow a characteristic course by first turning towards the dorsal body surface and then recurving ventrad and, subsequently, opening through the dorsal wall of the seminal vesicle. This specific course of the sperm ducts is uncommon among species of *Dugesia* and thus far had been reported only from *D. bengalensis*. However, in other features *D. bengalensis* is rather different from both *D. semiglobosa* and *D. majuscula*. For example, in *D. bengalensis* the sperm ducts open into the anterior portion of the seminal vesicle, while the ejaculatory duct has a subterminal ventral opening and the oviducal openings are symmetrical (Kawakatsu et al. 1983).

### Karyology

Within the genus *Dugesia*, the basic chromosome number is seven, eight, or nine (Stocchino et al. 2004). With respect to karyotypes with a haploid number of n = 8, there are species in which all chromosomes are metacentric and species in which some chromosomes are not metacentric. Since in *D. semiglobosa* and *D. majuscula* all chromosomes are metacentric, we shall restrict our discussion to those species that also exhibit chromosome complements with a basic number of eight, metacentric chromosomes. Such a chromosome portrait is present in the following species: *D. gonocephala* (Dugès, 1830), many Sardinian populations of *D. benazzii* Lepori, 1951, *D. etrusca labronica* Lepori, 1950, *D. sagitta* Schmidt, 1861, *D. elegans*, *D. indonesiana* Kawakatsu, 1973, *D. japonica*, and presumably also in *D. colapha* Dahm, 1967 (cf. Dahm 1967; Benazzi and Gourbault 1975; Kawakatsu et al. 1976; Ball 1979; De Vries 1984; Deri et al. 1999; Pala et al. 1999; Stocchino 2018). Evidently, none of these species is anatomically similar to *D. semiglobosa* or *D. majuscula*, otherwise we would have included these in our comparative morphological discussions (see above).

In view of the fact that both *D. semiglobosa* and *D. majuscula* exhibited mixoploid chromosome complements, it is interesting to note that such a combination of diploid and triploid metacentric chromosomes in a basic set of eight chromosomes has been reported also for *D. japonica* Ichikawa & Kawakatsu, 1964 and *D. siamana* Kawakatsu, 1980 (Oki et al. 1981; Stocchino et al. 2004 and references therein). Mixoploid complements have been reported also for other species of *Dugesia* with a different basic number of chromosomes, such as n=7 or n=9, which we shall here not further discuss.

It has been suggested that polyploidization is adaptive in harsh climatic conditions and extreme environments (Stebbins 1971). In a study on the freshwater planarians *Phagocata vitta* Dugès, 1830, *Polycelis felina* Dalyell, 1814, and *Crenobia alpina* Dana, 1776, Lorch et al. (2016) reported that mean chromosome numbers increased with latitude, with higher latitudes coinciding with harsher climatic conditions. In contrast, mean chromosome numbers decreased with increasing temperature, temperature ranges, precipitation, and net primary production. Furthermore, populations with high chromosome numbers, due to polyploidization, tended to reproduce asexually, whereas populations with relatively low ploidy levels reproduced sexually. Nevertheless, these results and conclusions may not be well applicable to the two new Chinese *Dugesia*’s, since both species are mixoploid and possess hyperplasic ovaries. It is highly likely that the hyperplasic ovaries in the sexualized, ex-fissiparous animals prevented normal reproduction. In such abnormal ovaries the oocytes are also anomalous (Harrath et al. 2014), thus preventing regular oogenesis. That the cause of the infertility lies in the hyperplasic ovaries is supported by the fact that the testes are well-developed and that, thus, spermatogenesis presumably is regular. That gonadic anomalies effectuate infertility is known also from other species of *Dugesia* (cf. Stocchino et al. 2012; Harrath et al. 2017). Nevertheless, it is known that in some species of *Dugesia* ex-fissiparous individuals are able to produce fertile cocoons (Harrath et al. 2013; Stocchino and Manconi 2013 and references therein; Stocchino et al. 2014) but, apparently, *D. semiglobosa* and *D. majuscula* do not have that capacity.

The fact that animals from both species sexualized under laboratory conditions suggests that these conditions induced, or at least were favourable to sexualization. In our laboratory cultures we used a lower temperature than that of their habitat, in that in our cultures water temperature was 20 °C, whereas temperature in the springs was 23 °C and 19 °C. Furthermore, the specimens were collected in the coldest month of the year, viz., February. In Hainan Island, the average temperature during the coldest period usually is 18–22 °C, a situation that lasts for ~ 40 days. However, the temperatures in other months of the year are considerably higher than the temperature at which we collected our specimens. Especially, average temperatures from May to October reach 26–35 °C, with the highest temperature frequently being higher than 39 °C. This means that most of the time the two new species experience a relatively warm environment, in contrast to the relatively cold laboratory cultures. At collection, none of the specimens exhibited any signs of reproductive organs, while in the laboratory they sexualized at a lower temperature than they usually experience in their natural surroundings, with *D. semiglobosa* even producing cocoons, albeit inviable ones. This suggests that the sexualization process was induced by the lower temperatures that the worms experienced in the laboratory.

## Supplementary Material

XML Treatment for Dugesia semiglobosa

XML Treatment for Dugesia majuscula

## Supplementary materials

Supplementary material 1 Figure S1. Phylogenetic tree obtained from ML analysis of the concatenated dataset Data type phylogenetic tree Explanation note Numbers at nodes indicate support values (bootstrap). Scale bar: substitutions per site. https://binary.pensoft.net/file/528478This dataset is made available under the Open Database License (http://opendatacommons.org/licenses/odbl/1.0/). The Open Database License (ODbL) is a license agreement intended to allow users to freely share, modify, and use this Dataset while maintaining this same freedom for others, provided that the original source and author(s) are credited.Lei Wang, Zi-mei Dong, Guang-wen Chen, Ronald Sluys, De-zeng Liu

Supplementary material 2 Table S1. Primers used for amplification, with their annealing temperatures Data type molecular data https://binary.pensoft.net/file/528479This dataset is made available under the Open Database License (http://opendatacommons.org/licenses/odbl/1.0/). The Open Database License (ODbL) is a license agreement intended to allow users to freely share, modify, and use this Dataset while maintaining this same freedom for others, provided that the original source and author(s) are credited.Lei Wang, Zi-mei Dong, Guang-wen Chen, Ronald Sluys, De-zeng Liu

Supplementary material 3 Table S2. Genetic distances for COI Data type molecular data Explanation note Highest and lowest distance values between the two new Chinese species and Oriental-Australasian congeners indicated in blue and red, respectively. Purple: distance value between the two new species. https://binary.pensoft.net/file/528480This dataset is made available under the Open Database License (http://opendatacommons.org/licenses/odbl/1.0/). The Open Database License (ODbL) is a license agreement intended to allow users to freely share, modify, and use this Dataset while maintaining this same freedom for others, provided that the original source and author(s) are credited.Lei Wang, Zi-mei Dong, Guang-wen Chen, Ronald Sluys, De-zeng Liu

Supplementary material 4 Table S3. Genetic distances for ITS-1 Data type molecular data Explanation note Highest and lowest distance values between the two new Chinese species and Oriental-Australasian congeners indicated in blue and red, respectively. Purple: distance value between the two new species. https://binary.pensoft.net/file/528481This dataset is made available under the Open Database License (http://opendatacommons.org/licenses/odbl/1.0/). The Open Database License (ODbL) is a license agreement intended to allow users to freely share, modify, and use this Dataset while maintaining this same freedom for others, provided that the original source and author(s) are credited.Lei Wang, Zi-mei Dong, Guang-wen Chen, Ronald Sluys, De-zeng Liu
