# Supplementary figures and images for: Integrative descriptions of two new species of Dugesia from Hainan Island, China (Platyhelminthes, Tricladida, Dugesiidae)
Source: Zookeys. 2021 Apr 5;1028:1–28. doi: 10.3897/zookeys.1028.60838 (PMC8041735; doi:10.3897/zookeys.1028.60838)

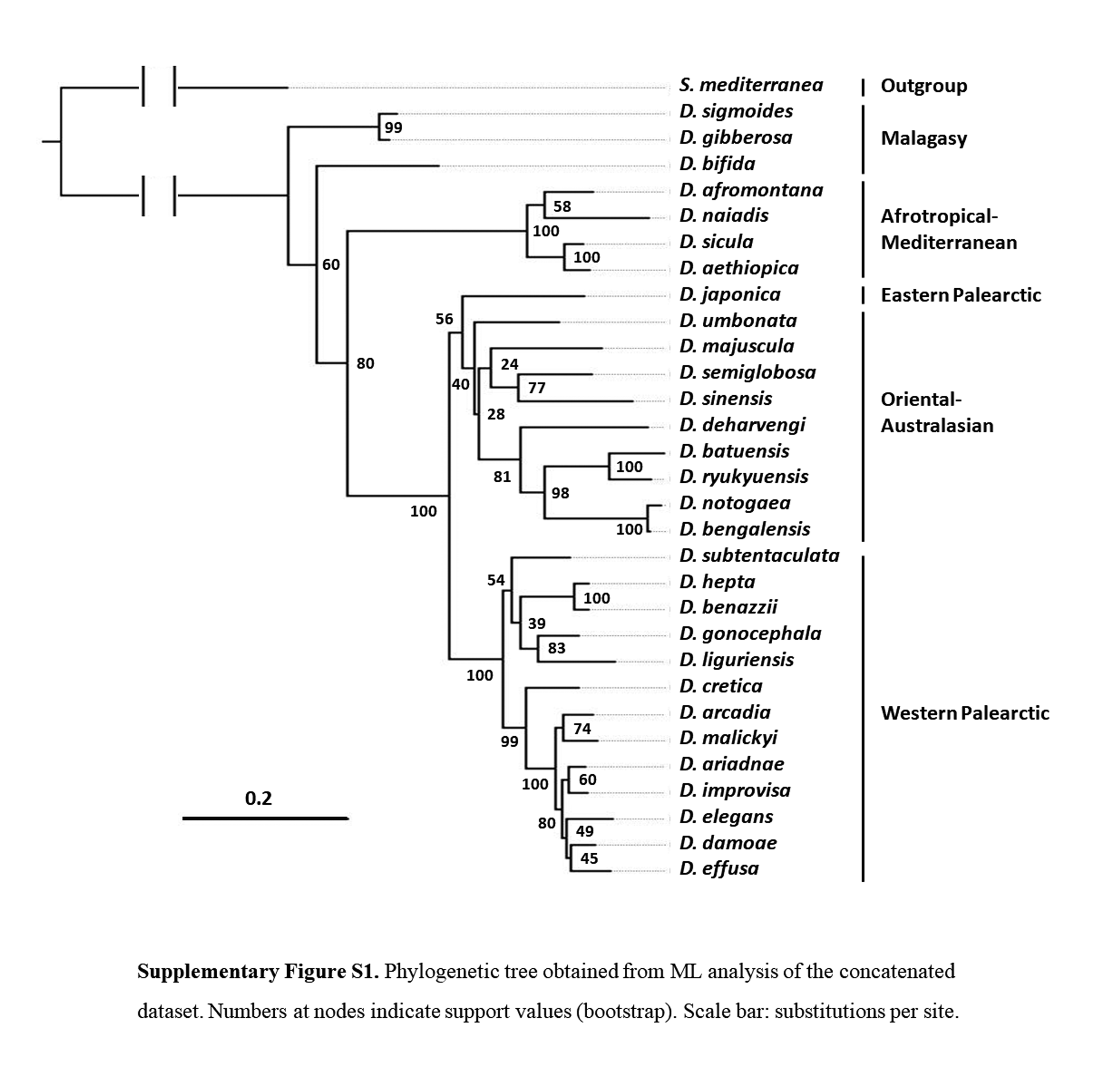

Supplement: Supplementary material 1 — Figure S1. Phylogenetic tree obtained from ML analysis of the concatenated dataset [file zookeys-1028-001-s001.tif]
