# Supplementary material for: Integrative descriptions of two new species of Dugesia from Hainan Island, China (Platyhelminthes, Tricladida, Dugesiidae)
Source: Zookeys. 2021 Apr 5;1028:1–28. doi: 10.3897/zookeys.1028.60838 (PMC8041735; doi:10.3897/zookeys.1028.60838)
Supplement: Supplementary material 2 — Table S1. Primers used for amplification, with their annealing temperatures [file zookeys-1028-001-s002.docx]

**Supplementary Table S1.** Primers used for amplification, with their annealing temperatures.

| Primer | Direction | Sequence 5–3 | Annealing temperature (°C) | References |
| --- | --- | --- | --- | --- |
| *Cox1*  COI F  COI R | F  R | ATAGGTGGKTTTGGTAAT  ATTWAYAWCAACACTACGAC | 44  44 |  |
| *ITS-1*  ITS 9F  ITS R | F  R | GTAGGTGAACCTGCGGAAGG  TGCGTTCAAATTGTCAATGATC | 45  45 | Baguñà et al., 1999  Baguñà et al., 1999 |

**Reference**

Baguñà J, Carranza S, Pala M, Ribera C, Giribet G, Arnedo M, Ribas M, Riutort M (1999) From morphology and karyology to molecules. New methods for taxonomical identification of asexual populations of freshwater planarians. Italian Journal of Zoology 66: 207–214. https://doi. org/10.1080/11250009909356258
