# Supplementary material for: Integrative descriptions of two new species of Dugesia from Hainan Island, China (Platyhelminthes, Tricladida, Dugesiidae)
Source: Zookeys. 2021 Apr 5;1028:1–28. doi: 10.3897/zookeys.1028.60838 (PMC8041735; doi:10.3897/zookeys.1028.60838)
Supplement: Supplementary material 3 — Table S2. Genetic distances for COI [file zookeys-1028-001-s003.docx]

**Supplementary Table S2.** Genetic distances for COI. Highest and lowest distance values between the two new Chinese species and Oriental-Australasian congeners indicated in blue and red, respectively. Purple: distance value between the two new species.

|  | *S. mediterranea* | *D. sinensis* | *D. majuscula* | *D. semiglobosa* | *D. notogaea* | *D .japonica* | *D. batuensis* | *D. deharvengi* | *D. ryukyuensis* | *D. umbonata* |
| --- | --- | --- | --- | --- | --- | --- | --- | --- | --- | --- |
| *S. mediterranea* |  |  |  |  |  |  |  |  |  |  |
| *D. sinensis* | 0.3951 |  |  |  |  |  |  |  |  |  |
| *D. majuscula* | 0.3946 | 0.1884 |  |  |  |  |  |  |  |  |
| *D. semiglobosa* | 0.3594 | 0.1408 | 0.1952 |  |  |  |  |  |  |  |
| *D. notogaea* | 0.3803 | 0.1837 | 0.1754 | 0.1662 |  |  |  |  |  |  |
| *D. japonica* | 0.4671 | 0.2360 | 0.2202 | 0.2106 | 0.2229 | 0.1990 |  |  |  |  |
| *D. batuensis* | 0.3746 | 0.2007 | 0.1572 | 0.1426 | 0.1784 |  |  |  |  |  |
| *D. deharvengi* | 0.3573 | 0.1828 | 0.1368 | 0.1630 | 0.1735 |  | 0.1622 |  |  |  |
| *D .ryukyuensis* | 0.3065 | 0.2003 | 0.1827 | 0.1460 | 0.1501 |  | 0.1383 | 0.1636 |  |  |
| *D. umbonata* | 0.3637 | 0.1470 | 0.1613 | 0.1061 | 0.1573 | 0.1799 | 0.1780 | 0.1471 | 0.1465 |  |
