# Supplementary material for: Integrative descriptions of two new species of Dugesia from Hainan Island, China (Platyhelminthes, Tricladida, Dugesiidae)
Source: Zookeys. 2021 Apr 5;1028:1–28. doi: 10.3897/zookeys.1028.60838 (PMC8041735; doi:10.3897/zookeys.1028.60838)
Supplement: Supplementary material 4 — Table S3. Genetic distances for ITS-1 [file zookeys-1028-001-s004.docx]

**Supplementary Table S3.** Genetic distances for ITS-1. Highest and lowest distance values between the two new Chinese species and Oriental-Australasian congeners indicated in blue and red, respectively. Purple: distance value between the two new species.

|  | *S. mediterranea* | *D. deharvengi* | *D. batuensis* | *D. ryukyuensis* | *D. bengalensis* | *D. notogaea* | *D. japonica* | *D. majuscula* | *D. semiglobosa* | *D. umbonata* |
| --- | --- | --- | --- | --- | --- | --- | --- | --- | --- | --- |
| *S. mediterranea* |  |  |  |  |  |  |  |  |  |  |
| *D. deharvengi* | 0.5034 |  |  |  |  |  |  |  |  |  |
| *D. batuensis* | 0.4795 | 0.1080 |  |  |  |  |  |  |  |  |
| *D. ryukyuensis* | 0.4933 | 0.1214 | 0.0269 |  |  |  |  |  |  |  |
| *D. bengalensis* | 0.4633 | 0.1236 | 0.0962 | 0.0927 |  |  |  |  |  |  |
| *D. notogaea* | 0.4719 | 0.1336 | 0.1039 | 0.0983 | 0.0107 |  |  |  |  |  |
| *D. japonica* | 0.4742 | 0.1019 | 0.1062 | 0.1152 | 0.1218 | 0.1361 |  |  |  |  |
| *D. majuscula* | 0.4617 | 0.0937 | 0.1058 | 0.1233 | 0.1132 | 0.1252 | 0.0582 |  |  |  |
| *D. semiglobosa* | 0.4713 | 0.1079 | 0.1350 | 0.1445 | 0.1451 | 0.1574 | 0.0974 | 0.0583 |  |  |
| *D. umbonata* | 0.4900 | 0.1207 | 0.1269 | 0.1320 | 0.1283 | 0.1340 | 0.0879 | 0.0838 | 0.1201 |  |
